# Decision making in the oesophageal cancer trajectory a source of tension and edginess to patients and relatives: a qualitative study

**DOI:** 10.1080/17482631.2023.2170018

**Published:** 2023-02-02

**Authors:** Malene Kaas Larsen, Helen Schultz, Michael Bau Mortensen, Regner Birkelund

**Affiliations:** a Department of Surgery, Odense University Hospital, Odense, Denmark; b Institute of Clinical Research, University of Southern Denmark, Odense M, Denmark; c Health Services Research Unit, Lillebaelt Hospital, Vejle, Denmark; d Institute of Regional Health Research, University of Southern Denmark, Odense M, Denmark

**Keywords:** Esophageal cancer, decision-making, qualitative research, patients’ experiences, relatives’ experiences, participant observations

## Abstract

**Purpose:**

The curative oesophageal cancer continuum—diagnosis, treatment and survivorship represents different phases with its own challenges for the involved parties. The process of treatment decisions and interactions between patients with oesophageal cancer (EC), relatives and health professionals is vital yet not well described. The purpose of the study was to explore patients’ and relatives’ experiences with the process of decision-making through the EC illness and treatment trajectory.

**Methods:**

Longitudinal explorative design was employed based on ethnographic fieldwork in the form of participant observations inspired by the American anthropologist James Spradley.

**Results:**

Sixteen patients and 18 relatives were recruited for participant observations. In total, 184 hours of participant observations were conducted. The study showed that decision-making was filled with tension and edginess. Four themes were identified: 1) The encounter with the medical authority, 2) The need to see the big picture in the treatment trajectory, 3) A predetermined treatment decision, and 4) Meeting numerous different health professionals.

**Conclusion:**

The EC trajectory and decision-making were filled with anxiety. Patients and relatives lacked an overview of the treatment pathway, leading to their role in decision-making often being governed by the medical authority. Timing information and continuity are vital factors in decision-making.

## Introduction

Oesophageal cancer (EC) is an aggressive, physically, and emotionally devastating illness with one of the poorest survival rates among all malignant tumours (Djarv & Lagergren, 2012). The worldwide survival of patients with resectable EC remains poor, with a three-year survival of 23–58% despite improvements in treatment over the past 20 years (Lagergren et al., 2017). Studies identify that patients and relatives deal with the shock of diagnosis and try to manage and get acquainted with a complex and efficient healthcare system in which tests and treatments are carried out quickly (Fletcher et al., 2017; Larsen et al., 2020, 2021; Thaysen et al., 2019). In many Western countries, the time between diagnosis and the start of treatment has been significantly reduced (Lagergren et al., 2017; Mansour et al., 2017), meaning patients and relatives do not have much time to consider and prepare for the new situation. In healthcare, as well as in the political agenda, patient-centeredness, patient participation, and shared decision-making (SDM) are considered crucial to ensure that patients are involved in their treatment and care (Coulter, 2011; Coulter et al., 2017; Thaysen et al., 2019).

Patients’ and relatives’ involvement is essential within the context of a life-threatening illness such as EC. Moreover, studies describe that the more fear patients have of EC recurring, the more information they want about the prognosis (Franssen et al., 2009; Mazor et al., 2013). Studies also describe that patients want more information about long-term conditions than health professionals expect and provide (Blazeby et al., 2015; McNair et al., 2016; Smets et al., 2012). Regarding the decision-making process, studies show that most patients prefer to be part of decisions, describing the importance of establishing a trusting relationship with health professionals (Chawla & Arora, 2013; McKneally & Martin, 2000). However, the challenge for health professionals is recognizing whether patients and relatives are ready to receive information and to what extent. Coulter (Coulter, 2011) contends that it is imperative to involve patients in decisions at pivotal times of change related to a need to modify a treatment plan, try a different medication, or plan for discharge. Sharing information is essential for patient participation and is a core theme in patient-centred care (Kitson et al., 2013). Given the poor prognosis of EC and the impact of surgery, it is crucial to address patients’ and relatives’ information and support needs. This can help patients and relatives regain control, reduce their anxiety, improve compliance, create realistic expectations, promote self-care, and generate feelings of security (Henselmans et al., 2012).

A cornerstone of patient-centred care is SDM, where health professionals and patients work together to reach a shared healthcare choice (Coulter et al., 2015, 2017). Shared decision-making is a core element of the more general concept of patient engagement and is a treatment decision model that prescribes how healthcare decisions should be made (Steffensen, 2019). The model is based on the idea that the healthcare professional communicates medical knowledge to the patients, and the patient’s perspectives, preferences, and medical options are included in the clinical conversation. Shared decision-making thus helps patients and their relatives gain more influence and participation in treatment decisions (Steffensen, 2019).

Patients with EC and their relatives have different experiences and challenges at different periods in the illness- and treatment course. Studies describe patients’ experiences and challenges with adapting to the diagnosis, treatment and everyday life (Andreassen et al., 2007; Bull et al., 2019; Hellstadius et al., 2019). Likewise, relatives are affected by the poor prognosis of EC, the effect of treatment, and the changed interrelationship between patients and relatives (Bull et al., 2019; Haj Mohammad et al., 2015; McCorry et al., 2009). Studies show that patients, relatives, and health professionals acknowledge the patients’ authority to have the final say over the decisions. However, it is recognized that relatives deserve to participate in decision-making because cancer diagnosis and treatment also affect them (Bracher et al., 2020; Shin et al., 2017). The process of decisions and interactions between patients, relatives, and health professionals and how patients and relatives experience decision-making is not well described. During patients’ EC illness and treatment, decision-making is a continuous process between patients, relatives, and health professionals based on mutual evaluation of how the treatment affects the patient.

Nuanced insight into the process of decision-making through the EC illness and treatment is needed. Extensive understanding based on a continuous period might provide valuable insight into patients’ and relatives’ evolving experiences. This understanding is essential in developing and implementing person-centred healthcare and decision-making. Therefore, the study aimed to explore patients’ and relatives’ experiences with the process of decision-making through the EC illness and treatment trajectory.

## Method

### Design

To gain a deeper understanding of decision-making, the methodological approach was based on ethnographic fieldwork in the form of participant observations inspired by the American anthropologist James Spradley (Spradley, 2006). A longitudinal explorative design was chosen to explore experiences over time among patients with EC and their relatives and explore the decision-making context. The longitudinal design enabled the exploration of development, patterns, and experiences from the same group of participants over time. Moreover, it enabled an understanding of possible changes in participants’ perspectives during illness and treatment.

### Setting

In Denmark, treatment for EC is regarded as a highly specialized treatment and is organized in four university hospitals. Curative treatment typically consists of three series of perioperative chemotherapy, followed by surgery, and after a recovery period, treatment with three additional cycles of chemotherapy. This is in line with international treatment protocols (Lagergren et al., 2017). After diagnosing EC, a multi-disciplinary team concludes whether or not the patient is offered curative treatment, considering the patient’s physical condition. The patient is informed about this conclusion afterwards. Patients are subsequently referred to the Department of Oncology as outpatients to start treatment with chemotherapy. After chemotherapy and a recovery period, patients are admitted to the Department of Surgery for operation. The admission for the operation has an average length of between 7–10 days. Then, patients have a short recovery period before they are appointed to postoperative chemotherapy.

### Recruitment and participants

The first author (MKL) recruited participants between March to November 2017. The inclusion criteria were: Danish-speaking patients over 18 years of age with biopsy-confirmed EC (adenocarcinomas) planned to undergo surgery and oncological treatment. Inclusion criteria for relatives were: Danish-speaking relatives over 18 years of age with a solid attachment to the patient, meaning they attended consultations and were present during the patients’ admission for surgery. However, one patient had no relatives participating in consultations or ward rounds. Patients and relatives were recruited by MKL during their first consultation about the treatment plan. No participants declined to participate.

### Data collection

**Figure 1.**
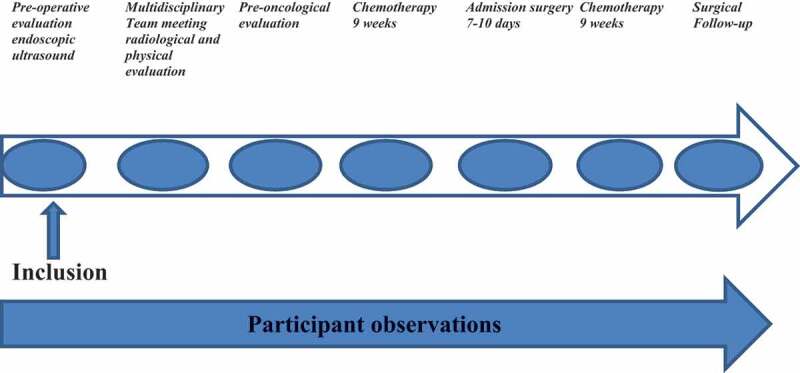
Overview of participant observations.

The first author (MKL) conducted participant observations from March 2017 to September 2018 at the oncological and surgical departments during outpatient consultations and surgery admission, where treatment decisions were expected to occur. MKL is an experienced researcher and clinical nurse specialist in the Department of Surgery, although not in the studied units. The observations covered the period before and during the treatment until the check-up consultation six months after surgery. The observations included conversations with patients and relatives about their experiences, thoughts and reflections regarding the EC illness and treatment trajectory and the decision-making process. Figure 1 shows an overview of participant observations during the treatment course. In total, 184 hours of participant observations were conducted. The focus of participant observation included:

- What was the context surrounding the process of decision-making?
- How did patients and relatives express themselves over time in the context of EC treatment and decision-making?
- How did health professionals involve patients and relatives in treatment and decision-making?
- What was the interaction like between the patient, relative and health professional?

According to Spradley and Emerson, descriptive field notes, including direct quotations, were made during and after the observations and transcribed afterwards (Emerson et al. 2011; Spradley, 2006). Spradley’s nine dimensions were used as a framework containing: space, actor, activity, object, act, event, time, goal, and feeling, ensuring a systematic approach to participant observations (Spradley, 2006).

## Data analysis

Data were analysed and interpreted with the inspiration of Ricoeur’s theory of interpretation, entailing the analysis consisted of three steps: a naïve reading, a structural analysis, and a critical interpretation (Ricoeur, 1976, 1984; Simony et al., 2018). In the naïve reading, the transcribed field notes were read several times to grasp an overall understanding of the decision-making context. According to Ricoeur, this initial reading should be based upon an open approach towards how the text moves and affects you, which leads to the first initial understanding (Ricoeur, 1976, 1984).

Following the naïve reading, a structural analysis was conducted. The structural analysis is an explanatory procedure that opens the text for further interpretation, focusing on units of meaning across the data based on “what is said” (Ricoeur, 1976). This refers to quotes that illuminate meanings and represent the data. Through further interpretation, units of significance were identified as descriptions of “what the text talks about.” In the process of structuring units of meaning and units of significance and extracting themes, the analysis moves forward and backward. Ricoeur describes this as a process of dynamic interpretative reading (Ricoeur, 1976). Final themes were interpreted from units of significance. Table 1 illustrates an example of the structural analysis.

**Table I.** Example of the systematic process in the structural analysis.

Units of meaning (what is said/observed)	Units of significance (what the text speaks about)	Theme
”I read in my electronic journal. You need to see the big picture and be in control of the situation. Seeing the big picture is very important to me”. (Department of Surgery, Patient 6 & Relative 7)	Establishing an overview by reading in the electronic journal	**Patients’ and relatives’ need for seeing the big picture in the treatment trajectory**
The wife asks about her husbands’ chances for survival. (Department of Surgery, Patient 10 & Relative 13)	Thinking and asking about the worst-case scenario	
The patient tells the oncologist that he appreciates the explanation about the time after surgery before starting the chemotherapy. He says he now has created an overview of the entire treatment plan. (Department of Oncology Patient 1 & relative1)	An overview of the entire treatment plan	

In the critical interpretation and discussion, the authors then interpreted and discussed the themes with relevant theoretical concepts and research results. According to Ricoeur, the critical interpretation is an in-depth interpretation that points towards a possible world and seeks to understand and grasp a new understanding of the world (Dreyer & Pedersen, 2010; Ricoeur, 1976, 1984).

## Ethics

The study was conducted in accordance with the Declaration of Helsinki (Association, 2013) and approved by the Danish Data Protection Agency (ID No. 16/155593). The regional health research ethics committee was informed and ruled that the study required no ethical approval. The participants received oral and written information about the study and signed informed written consent. Participant observations were handled with respect and consideration for patients, relatives and health professionals, always acknowledging their well-being. To ensure participants’ anonymity, we obscured all personal characteristics in this article. Thus, we refer to a small population in a specific geographical region (Morse & Coulehan, 2015).

## Researcher reflexivity and trustworthiness

MKL conducted the recruitment and participant observations. MKL is an experienced researcher and surgical nurse who works in the Department of Surgery, although not in the units studied. During observations, the nine areas in Spradley’s observation toll guided a systematic focus and documentation of the observed situations (Spradley, 2006). In the field notes, the researcher carefully distinguished between objective observations and personal reflections, as recommended by Emerson (Emerson RIFLLS, 2011). The interpretation of participant observations was discussed with the authors, who all are experienced researchers. The analysis was conducted in close cooperation with the second and last author to ensure trustworthiness. The participants were used in reflections regarding interpretations of shared situations during participant observations. However, they were not asked to validate the findings. According to Ricoeur, the narrative of our experiences will always be a reflection on what happened, and once told, new reflections will emerge with new perspectives that change the experiences (Ricoeur, 1984).

## Results

### Participant characteristics

Sixteen patients and 18 relatives were recruited for participant observations from March to November 2017. Fourteen out of 16 patients were men, causing most relatives to be wives. Tables 2 & 3 show the characteristics of patients and relatives.

**Table II.** Characteristics of the patients.

Total of 16 patients	Number
**Age, years**	
45–60	2
60–70	8
70–80	6
**Gender**	
Male	14
Female	2
**Living with a partner**	13
**Employed**	4
**Retired**	12
**Did not get planned surgery**	3
**Received the intended curative treatment**	9
**Did not complete the postoperative chemotherapy**	4

**Table III.** Characteristics of the relatives.

Total of 19 relatives	Number
**Age, years**	
20-49	4
50-59	3
60-69	8
70-79	4
**Gender**	
Male	2
Female	17
**Relation**	
Spouses	15
Daughter, granddaughter, brother-in-law, sister	4
**Employed**	11
**Retired**	8

## Main results

The naïve reading and structural analysis revealed that the context of decision-making throughout the EC trajectory was filled with tension and edginess. Patients and relatives needed to establish an understanding of the treatment course and its effect before engaging in treatment decisions. Four themes were identified: 1) The encounter with the medical authority, 2) Patients’ and relatives’ need to see the big picture in the treatment trajectory, 3) A predetermined treatment decision, and 4) Meeting numerous different health professionals. In the next section, we explore these themes in more detail.

## The encounter with the medical authority

When the patient had finished the diagnostic work, patients and relatives were invited to a consultation with the surgeon about possible treatment. Part of this consultation was for the surgeon to assess the patient’s physical ability to undergo the extensive oesophageal surgery and neoadjuvant chemotherapy and subsequently discuss possible treatment. If the surgeon judged the patient as physically capable of receiving treatment with curative intent, the patient and relatives were presented with a treatment plan. The observations showed that the surgeons addressed considerations regarding the patient’s physical capability for surgery in an open manner, as shown in the following field note:

The surgeon tells the patient that the operation entails putting the patient under general anaesthesia from eight am to three pm. Furthermore, the operation and subsequent rehabilitation demand physical strength and typically last up to six months. Therefore, the patient needs to be in good physical shape. The surgeon gives the information in a high and authoritarian voice. (Department of Surgery, patient 1 & relative 1)

Patients and relatives were aware that the patient’s capability for treatment was assessed by surgeons and felt privileged if the patient passed this eye of the needle, meaning there was hope for a cure and a future without cancer.

The assessment of whether the patients were capable of treatment was evident in the surgical and oncological departments. In the Department of Oncology, the assessment was pointed towards possible side effects of the chemotherapy. Therefore, it was essential to instruct the patients and relatives about reacting to possible side effects like fever, diarrhoea and nausea at home. Observations showed that decision-making about possible modifications in chemotherapy due to side effects was a delicate issue as the patients and relatives often were uncomfortable with not following the planned treatment, constantly thinking about the prognosis if changes to the treatment plan were made:

The oncologist asks the patient about the side effects of chemotherapy. The patient minimizes side effects and says he is not bothered by small things. The oncologist asks for details, and the patient replies that he has a prickly sensation in his feet and has nausea. The nurse asks if the prickly sensation is present now, seems alarmed, and informs the patient about the possibility of a chronic condition if the chemotherapy proceeds. The patient replies, “Well, it is not that bad. I think we shall proceed with the planned last series with chemotherapy. I prefer we follow the plan”. (Department of Oncology, patient 6 & relative 7)

Patients and relatives were aware of the bad prognosis for EC, so they saw it as essential to follow the entire treatment plan. Therefore, within the context of a life-threatening illness, some patients minimized side effects and even tried to conceal if they were affected by the chemotherapy, meaning decision-making about chemotherapy was filled with tension and edginess.

However, observations also showed that patients and relatives could find they lacked knowledge for decision-making, leading to relief when the medical authority took the initiative and verbalized their opinions and preferences, as shown in the following field note:

The oncologist tells the patient that there are two options; to reduce the doses of chemotherapy to 75% or refrain from giving one out of three chemotherapy substances, awaiting the patient’s answer. The patient replies, “I find this decision hard to make, but I am not interested in persistent reduced sensation in my feet and fingers.” The oncologist says she thinks they must reduce the doses of chemotherapy and refrain from giving one chemotherapy substance. The patient says yes, and seems pleased with the oncologist making the decision. After the consultation, the patient tells the observer that it was nice that the oncologist made the decision and that he needed the oncologist to take authority in the decision-making. (Department of Oncology, patient 4 & relative 5)

Patients and relatives acknowledged and appreciated surgeons’ and oncologists’ medical authority because they felt vulnerable and insecure in the EC trajectory. Participant observations showed that relatives’ role was of varying extents, often contingent on an invitation from health professionals to participate in decisions and contingent on an invitation from the patient.

## Patients’ and relatives’ need to see the big picture in the treatment trajectory

As the treatment period for EC was long and typically consisted of three series of chemotherapy, surgery, and after a recovery period, treatment with three series of chemotherapy again, patients and relatives needed to see the big picture in the treatment trajectory from the beginning. Observations showed that patients often read the electronic journal to create an overview and insight into the illness, asking detailed questions about what they had read in their journal. However, the health professionals often gave information about the entire treatment to a limited extent, neglecting patients’ and relatives’ need for an overview, as shown here from a consultation before the start of treatment:

The surgeon tells the patient and relatives about the treatment course consisting of chemotherapy, surgery, and again chemotherapy. The patient asks for details about the operation regarding how the tumour is removed. The surgeon says that the operation is a laparoscopic procedure. Says then, “If you don’t mind, I prefer to put off further details about the operation until later. You start with the chemotherapy and cannot relate to details about the operation anyway. Direct his attention solely to the patient.” (Department of Surgery, Patient 7 & relatives 8 & 9)

The observations showed that patients and relatives did not pursue questions but merely acknowledged that health professionals were setting the agenda of meetings. Furthermore, relatives’ role in decision-making was very quiescent if they did not get an active request to participate from the health professionals. However, if health professionals called attention to relatives and incited their active participation, relatives asked detailed questions, striving for comprehension of the entire treatment course as shown here:

The young surgeon, engaged in medical specialization, asks if the patient and the wife have questions and looks inciting at them. The wife asks if the surgery entails the whole stomach is being removed or just a part of it. She says they haven’t got the full description of the operation, and she needs to know. The surgeon starts to look in the journal for a description. The patient says he thinks it has to do with the tumour’s position and doesn’t need these details. The patient says, “never mind; we will know this after the operation.” (Department of Surgery, Patient 1 & Relative 1)

Relatives were aware of the severity of the illness and were striving to understand the illness and its treatment, meaning they often asked detailed questions. However, observations showed that patients and relatives were not always agreeing with questions, sometimes causing conflicts between patients and relatives driven by different needs for information. These conflicting needs for information gave rise to relatives being in a state of bewilderment, trying to comprehend and embrace the new situation and their new role as relatives. Therefore, it was essential for relatives to adjust and reflect on the new situation with cancer in the mutual life, making it easier to navigate as relatives during the treatment course.

Timing of information was another issue addressed by the patients as they had experienced examples of situations with inappropriate information, as the following field note shows:

The patient tells the observer about a situation in connection with the operation. “Well, I was lying in my bed at the operation theatre when the surgeon came and told me that in 10% of the operations, they cannot do the planned procedure and remove the tumour. I do not need this information down at the operation theatre. This information should have been presented on an earlier date. I was lying there alone in the operating theatre, very nervous, awaiting the start of the operation.” (Department of Surgery, patient 8)

Patients and relatives strove to navigate the new situation. The timing of information about treatment and its consequences was essential as it helped them prepare and adjust to the new conditions. They appreciated straightforward communication but at an appropriate time. Furthermore, observations showed that health professionals often missed supporting patients and relatives with sufficient knowledge to participate in decision-making prompting patients’ and relatives’ anxiety and leading to a lack of influence on decisions for the patients and relatives.

## A predetermined treatment decision

The observations showed that health professionals often neglected to exchange knowledge and information about the treatment but merely presented them with what they thought was the best treatment plan. Patients’ and relatives’ perspectives and preferences were often not visible in consultations, and the patients and relatives strove to get acquainted with the agenda of the meeting. The speed in the time from diagnosis and the start of treatment entailed leaving patients and relatives with little time for reflection on decisions. Moreover, often the health professionals presented the plan for treatment for the patient and relative, emphasizing that cancer was a potentially lethal illness, as shown in the following field note:

The patient and the surgeon are having a conversation about the operation. The surgeon says, “As I see it, you do not have any choice. You die if we do not operate, so in my opinion, it is an easy choice. We have a chance of curing you.” (Department of Surgery, patient 8 & relative 10)

The health professionals presented patients and relatives with such categorical statements, leaving little or no time for deliberation, disregarding the opportunity for patients and relatives to reject the treatment plan. Sometimes the patient and relative asked questions about the prognosis and chances for a cure:

The wife asks what her husbands’ chances are. The surgeon replies, “This is a good question. It looks like the tumour is in an early stage. We will know more after the surgery, but if we don’t do anything, you will die in two years,” he looks at the patient. The patient asks about the survival rate, and the surgeon says that 25–30% of the total population of patients with EC survive more than five years treated or untreated. The patient and wife look despondent when hearing this. (Department of Surgery, patient 10 & relative 13)

Patients and relatives were aware that EC posed a threat. Consequently, they were always on guard, and it was essential for them to know about the long-term prognosis. This was a way of preparing their minds for the upcoming treatment and a foundation for making decisions. However, often health professionals did not provide much space or time for questions, as shown here from a conversation between the surgeon, patient and relative about gastroscopy conclusion and treatment options:

“Your tumour grows through the four layers in your oesophagus. I have also seen four lymph nodes, but nothing that implies we cannot operate. The treatment is chemotherapy for three months, then an operation. That is if you accept?” The patient says yes. The surgeon does not explain further or ask for questions but starts talking about a research project. (Department of Surgery, patient 14 & relative 17)

The missing invitation for questions made it difficult for patients and relatives to engage in decisions actively. Instead, their role in decisions was subordinate to the health professionals, meaning patients and relatives were unwillingly pushed into a position of powerlessness and vulnerability. Therefore, patients and relatives were not actively collaborating but merely bystanders in predetermined decisions.

Sometimes health professionals did have an open dialogue and asked for patients’ perspectives, as shown here:

The oncologist looks at the patient and asks, “What is your opinion on a new cycle of chemotherapy?” The patient asks if it would be wise to say yes. The oncologist tells the patient that he must be physically ready for a new cycle of chemotherapy. The conversation proceeds with the patient telling details about a decline in side effects of chemotherapy, like nausea. They mutually degree to continue the chemotherapy. (Department of Oncology, patient 12 & relative 15)

If the health professional invited the patient and relative to give their perspective on treatment, patients and relatives were actively engaged in treatment decisions, meaning that the decision was based on an increased information level for patients, relatives and health professionals.

## Meeting numerous different health professionals

As the treatment for EC typically consisted of treatment in different departments, patients and relatives met many different health professionals in the Department of Surgery and Department of Oncology. The observations showed that patients and relatives were more active in decision-making if they were familiar with health professionals throughout the treatment course. However, the patients and relatives got frustrated when they encountered too many different health professionals and different opinions, as shown here:

The patient tells the observer about his problems with pain management. Says, “In every shift, I have three nurses with three different opinions about how to relieve my pain. Well, it is nine nurses per day with different views and suggestions. It is frustrating as a patient, and I cannot handle so many people and opinions. I need a plan. (Department of Surgery, patient 10)

The number of health professionals gave rise to frustrations and mistrust and no opportunity to evaluate pain management continuously. As a result, the patients and relatives expressed that they became confused and filled with anxiety. Consequently, the patient was put in a position of passivity and bewilderment, leaving decisions to health professionals. Therefore, patients’ and relatives’ experiences and perspectives remained tacit and not included in the decision-making.

Observations showed that patients valued confidence and familiarity with health professionals, making them more at ease during the treatment. However, the treatment in different departments and the transition from one department to another gave rise to unanswered questions:

The patient has a consultation with the oncologist before starting the postoperative chemotherapy. The oncologist asks about the patient’s physical performance. The patient tells about his weight loss and difficulties at mealtimes, asking specific questions about his stomach pain. The oncologist neglects to answer but asks for the patient’s consent to chemotherapy. After the consultation, the patient tells the observer that he thinks it was not a supportive consultation but a waste of time. Says, “I think it was too much this, that and the other because I haven’t met him before, so he doesn’t know me or my problems. I can only hope he confers with the surgeon.” (Department of Oncology patient 4 & relative 5)

After surgery and discharge, patients were treated on an outpatient level, meaning they were responsible for reacting to symptoms and managing side effects from cancer treatment. Observations showed that patients and relatives often felt insecure and needed support from health professionals. Meeting health professionals who understood the problems in the EC treatment and who could support them was a cornerstone for patients and relatives. Patients and relatives appreciated that they were met in their differing needs throughout the continuum of the EC treatment by a minimum of health professionals and felt insecure and alone if this was not the case:

The patient tells the observer about his thoughts about the upcoming ward round at the admission for surgery. “I know I cannot expect the health professionals to be the same around me. But if they know me, they understand my problems and are more capable of my care. It gives me peace of mind that I am under surveillance from the same persons. Therefore, I hope it is one of the two surgeons who come on ward rounds.” (Department of Surgery, patient 8)

Through the participant observations, it became evident that patients and relatives were vulnerable, always alerted to how the treatment went on and the patient’s reactions to the prolonged treatment period. Encounters with familiar health professionals created an environment of safety and comfort in a distressing situation.

## Discussion

Our findings revealed the complexity for health professionals of balancing patients’ and relatives’ different stages of readiness towards decision-making. Patient-centred communication and building trust are vital in the process of decision-making. In the following, we will discuss preconditions in decision-making, patients’ and relatives’ roles in decision-making, and patient-centred communication as a fundamental aspect of decision-making.

This study illustrated that patients and relatives acknowledged and appreciated health professionals’ medical authority in decision-making and, to a large extent, acknowledged that health professionals set the agenda of meetings. A vital precondition in patients’ and relatives’ engagement in decision-making was a relationship with health professionals built upon acknowledgement of patients’ and relatives’ need to see the big picture in the treatment trajectory and the right timing of information. In addition, patients and relatives valued confidence and familiarity with health professionals. In this study, 14 out of 16 patients were men, causing most relatives to be wives. How men and women perceive and understand the EC illness can differ. According to Wenger and Oliffe, characteristics of men in Western culture include masculine ideals such as strength, control, competitiveness and performance (Wenger & Oliffe, 2014). Other studies describe that male cancer patients focus on the physical impact of the illness across the course of illness and treatment, most often employing a matter-of-fact approach, practicing trust and seeking to stay present (Handberg et al., 2015; Wenger & Oliffe, 2014). Regarding decision-making, there seem to be various reasons why patients and relatives respectively do not feel ownership towards the decisions regarding the treatment. Adding a gender perspective points to men practicing trust as a strategy and taking things one day at a time to balance control of their emotions, and the fear of losing control influences their participation in decisions.

The curative EC continuum—diagnosis, treatment, and survivorship represents different phases with its own challenges for patients and their relatives. This study revealed that patients and relatives emphasized the need for information outlining the entire course of treatment and the long-term effects of the treatment. Decision-making around treatment is described as a common point of a communication breakdown, as crucial decisions are often required during the earliest phase of illness when emotionality is intense, relationships are new, and information overload occurs (S. E. Thorne et al., 2005). Furthermore, Reyna et al. describe that cancer treatment decisions are complex as patients and relatives may wish to weigh the costs and benefits of treatment concerning the duration of symptom-free survival, time to relapse, and impact on quality of life functional status (Reyna et al., 2015). This study identified that health professionals often underestimated patients’ and relatives’ knowledge needs, leaving them puzzled and without an overview of their situation. The complexity of the decisions and this puzzlement made patients and relatives more or less inactive, leaving decisions to a great extent to the medical authority. Patients and relatives were aware that EC posed a threat leading to a need for knowledge about the long-term prognosis. This was a way of preparing themselves for the treatment and its consequences and was a foundation for decision-making. Thereby, the study supports other studies describing that patient participation is an active process, and several factors determine the degree of patient participation; the severity and type of illness the patient is experiencing, organizational structure, the patients’ amount of knowledge, and the desire to participate (Cahill, 1996; Thaysen et al., 2019).

This study identified that relatives’ role in decisions was of varying extent, often contingent on invitation from health professionals and the patient. Relatives asked detailed questions if the health professionals called attention to relatives and incited their active participation. Thereby the study supports other studies emphasizing that relatives’ participation in decision-making depends on the behaviour of the health professionals, culture, permission given by the organization, and the age and stage of relatives (Laidsaar-Powell et al., 2016; Partanen et al., 2018). Laidsaar-Powell et al. described in their study that a considerable amount of information gathering/exchange and deliberation occurred outside the consultation “behind the scenes” at home with family members on the sideline. Moreover, patients indicated that family involvement in decision-making was a benefit (Laidsaar-Powell et al., 2016). Thereby, addressing and inviting relatives’ engagement in decisions becomes even more essential. However, our study highlighted the complexity of balancing patients’ and relatives’ different stages of readiness in building knowledge about the EC trajectory while acknowledging relatives’ roles throughout the EC illness and treatment.

This study indicated that lack of continuity was a barrier to decision-making. Constantly meeting different health professionals gave rise to frustrations and mistrust. Consequently, the patients and relatives were put in a position of passivity and bewilderment, leaving decisions to health professionals. Epstein and Street have identified six critical functions of patient-centred communication in cancer care: fostering healing relationships, exchanging information, responding to emotions, managing uncertainty, making decisions, and enabling patient self-management (Epstein & Street, 2007). In this conceptual framework, decision-making is connected to other aspects of patient-centred communication, such as trust, which is a central part of fostering healing relationships, and patient self-efficacy, which is at the core of enabling patient self-management. For patients with EC and their relatives, it is a challenge to survey a treatment course consisting of surgery and perioperative chemotherapy (Larsen et al., 2020, 2021), and the duration and the effects of treatment add to the strain on patients and relatives (Andreassen et al., 2007). Therefore, building trust and a relationship with health professionals are essential as health professionals play a vital role in preparing and supporting patients and relatives.

Thorne et al. investigated barriers and facilitators to cancer communication in their qualitative study with 60 cancer patients. They found that full engagement in SDM requires that patients feel known by clinicians because they are distinguished as individuals and accommodated within the interactions and SDM (Thorne et al., 2013). However, studies show that decision-related uncertainty is complex and involves aspects of physician-patient risk communication, such as the capacity of treatment to improve patients’ health in the long term and the interpretation of statistics, especially when evidence and the patient’s understanding of given explanations are lacking (Kasper et al., 2008; McNair et al., 2016). Moreover, as Penrod points out, the experience of uncertainty is embedded in the individual’s sense of confidence and control, determining the nature of the experience of uncertainty (Penrod, 2007). Thereby, it becomes essential to address patients’ and relatives’ existential and situational modes of uncertainty before decision-making in ways they are comfortable. For this reason, there is a need for health professionals to tailor their SDM approach to patients and relatives and the decision context. Urgent or emergent decisions may not allow sufficient time for patients and relatives to engage in SDM (Malterud, 2001). However, our study illustrated that if health professionals engaged patients and relatives in decisions, they asked detailed questions, meaning the decision was based on increased understanding. Thus, patients’ and relatives‘ anticipation of the treatment course is essential. Still the challenge for patients and relatives of building realistic expectations may also be about building a new identity with a changed physical capability.

## Strengths and limitations

The study’s longitudinal design entailed that patients, relatives, and the researcher met throughout the treatment from diagnosis until six months postoperative. The prolonged data collection resulted in rich insights into the decision-making process and is considered a strength of the study. In addition, participant observations provided a unique opportunity to observe how patients and relatives were engaged in decision-making and whether the need for participation evolved during the treatment period. Throughout the study, patients and relatives expressed they felt grateful to be able to be treated with the aim of a cure. This position of gratitude may have influenced their interactions with health professionals in decisions. A limitation of the study is that observations were not performed during nights and weekends, which does not necessarily cover all decisions regarding treatment, especially not decisions made on unexpected incidents. The researcher’s presence will always affect the topic under investigation (Malterud, 2001; Spradley, 2006) and may have influenced the process of treatment decisions.

The study showed that some patients and relatives were gathering information from reading their electronic journals and the Internet, causing them sometimes to know more about test results than the health professionals. However, not all countries have electronic journals or open access to these journals, limiting the transferability in terms of how prepared patients and relatives can be and what amount of information they demand. Moreover, the study was conducted in a public healthcare setting in Denmark, which may limit its transferability to other countries with different healthcare environments. Furthermore, patients and relatives were recruited from a single hospital in Denmark, which may reflect local factors and conditions. However, no patients or relatives declined to participate in the study, which assisted in gathering detailed descriptions and illustrating diverse aspects of the continuum of participation in decisions, thus promoting a high degree of information power (Malterud et al., 2016).

Fourteen out of 16 patients were men in this study, causing most relatives to be wives. This is representative of the population, as men ≥60 years are overrepresented among patients with EC. The mean male-to-female ratio is 6:1 for patients with oesophageal adenocarcinoma (Lagergren et al., 2017). This may have impacted the experiences with decisions and how they reacted to the authority of health professionals. Possibly younger persons would ask to participate differently. In addition, a limitation is that only ethnic Danes participated in the study, causing minority groups not to be represented.

## Conclusion

The awareness of EC as a life-threatening illness entails the EC decision-making during treatment is filled with tension and edginess. Within the context of EC as a life-threatening illness, patients and relatives often see it as essential to follow the entire treatment plan, minimize side effects, and even conceal the effects of treatment. Patients and relatives experience the EC trajectory and decision-making with anxiety and a lack of overview of the treatment pathway, leading to their roles in decision-making often being governed by the medical authority. Moreover, patients and relatives are sometimes in conflict due to different information needs, inducing bewilderment and anxiety. Patients’ and relatives’ perspectives and preferences are often not visible in the decision-making as health professionals often miss supporting patients and relatives with sufficient knowledge to participate in decision-making. Especially the relatives’ role in decisions is tacit and often contingent on invitation from the health professionals. Timing information and continuity are vital factors in decision-making.

## Data Availability

To protect individuals’ privacy, data cannot be shared publicly.
